# Human Subconjunctival Dirofilariasis Presenting as the Daytime Photophobia: A Case Report

**Published:** 2017-10

**Authors:** Seyed Ali TABATABAEI, Mohammad SOLEIMANI, Bahram NIKMANESH, Raziyeh MAHMOUDZADEH, Zakieh VAHEDIAN, Mirataollah SALABATI, Zahra SOLEIMANI, Amir MATINI, Mahyar NOORBAKHSH

**Affiliations:** 1. Farabi Eye Hospital, Tehran University of Medical Sciences, Tehran, Iran; 2. Zoonosis Research Center, Tehran University of Medical Sciences, Tehran, Iran; 3. Dept. of Medical Laboratory Sciences, School of Allied Medical Sciences, Tehran University of Medical Sciences, Tehran, Iran; 4. Shahid Beheshti Hospital, Kashan University of Medical Sciences, Kashan, Iran

**Keywords:** Subconjunctival dirofilariasis, *Dirofilaria immitis*, Eye, Photophobia, Case report

## Abstract

We report a case of subconjunctival worm with a rare presentation of diurnal photophobia and temporal conjunctival injection. This case report describes a subconjunctival dirofilariasis in a 59-year-old man presented with foreign body sensation, localized tenderness, and eye redness during the day. After removal of subconjunctival 10 cm worm, the diagnosis was compatible with *Dirofilaria immitis*. Proof of identity was based on the morphological appearance, which were reliable diagnostic clues. Ocular examination was normal one month later except for faint temporal conjunctival scar. Subconjunctival dirofilariasis could present as diurnal photophobia and conjunctival injection.

## Introduction

Dirofilariasis is the member of Onchocercidae family and subfamily of Dirofilariinae of the Spirurida*.* This zoonotic infection is a universal concern. There have been different case reports from all over the world, approximately 782 cases up to now (1). The parasite in charge of this infection pollutes broad spectrum of animals involving predators like foxes, otters, sea lions, wolves, dogs and cats (2). Microfilariae are defined as the blood born stages of the parasite transferred by mosquito vectors called *Aedes* and *Culex* (3).

Human beings are accidental zoonotic infection targets in this infectious cycle. First of all the microfilariae is ingested by special kind of mosquitos mentioned earlier. The next step is the development and growth of larvae of the insect in order to become mature worms over two molts. In this level of maturity, the filariform larva is capable of spreading infection to special hosts through blood sucking mosquitoes (4).

*Dirofilaria* is the example of one the most common types of infection transmission. This filariform larva is caused by the dog heartworm. One of the common subtypes of *Dirofilaria* is known as *D. immitis*, which is responsible for pulmonary infections (5), and the spreading of this infection to other parts of body other than mentioned above is so rare and as a result, the involvement of the surrounding eye tissue is the least common site of infection (6). Involvement of subcutaneous tissue is another rare accident caused by *D. repens*. This site of involvement is rarely seen in human either. This blood sucking mosquitoes accidentally infect humans in this cycle (7).

There are two species of *Dirofilaria* including *D. immitis* and *D. repens* (8, 9) which are responsible for mentioned infection sites above but as we told earlier the involvement of ophthalmic tissue including eyelid, periorbital tissue, and intravitreal fluid are rarely reported (10) and the most interesting part is the infrequent subconjunctival tissue involvement (11).

Here we report a case of subconjunctival worm with a rare presentation of diurnal photophobia and temporal conjunctival injection.

## Case report

**Fig. 1:**
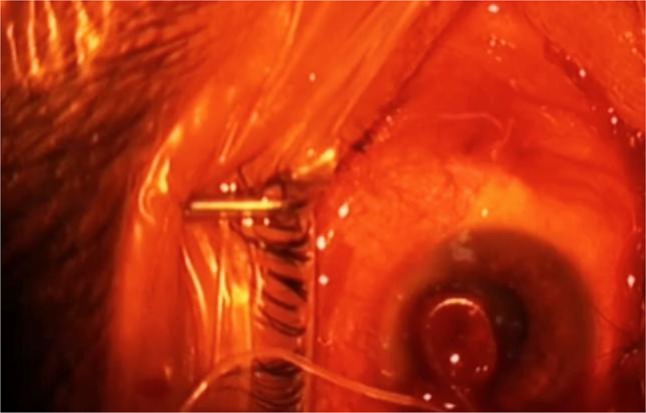
A 10 cm white intact roundworm was extracted from the conjunctiva

A 59-year-old overnight custodian was referred to emergency room with incremental complaint of diurnal foreign body sensation, localized tenderness and redness in his right eye since five days ago. He explained that the onset and worsening of these symptoms were because of extreme overnight fatigue led to foreign body sensation especially aggravated at the end of his work in the daylight. He also claimed his symptoms alleviated after sleeping but started at the end of his work the next day. He was referred by the occupational physician because of possible malingering. Ocular examination showed moderate chemosis and injection at that temporal conjunctiva. After careful slit lamp examination, a U-shaped moving lesion was visible under the conjunctiva at the site of redness and injection. In fact, when the light focused on the conjunctival surface, the worm started to move (Fig. 1).

**Fig. 2:**
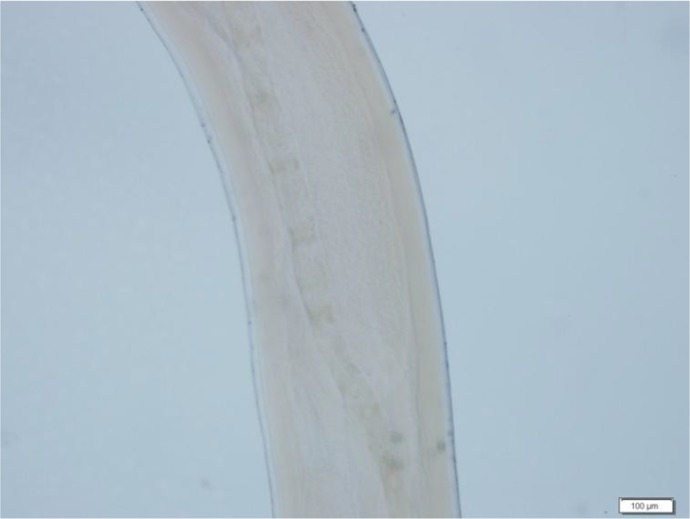
Smooth laminated cuticle, narrow hypodermal lateral cords and long muscle cells confirmed *Dirofilaria immitis*

Other ophthalmic examinations including visual acuity were normal. There was no history of recent travel, trauma, allergy or previous cutaneous lesions. Laboratory evaluation revealed normal blood cell count with a normal differential white blood cell count. There was no parasite in the stool specimen and the urine analysis was normal. The lesion was explored under topical anesthesia with 2% lidocaine using a localized peritomy. Finally, a 10 cm white intact roundworm was extracted from area and the specimen was sent to the Parasitology department for diagnosis. Extracted worm was reported to be an immature female worm with 100 mm length and 0.5 mm width belonging to *D. Immitis* family. Proof of identity was based on the morphological appearance and reliable diagnostic clues were completed with observing smooth laminated cuticle, narrow hypodermal lateral cords and long muscle cells (Fig. 2) on follow-up visits one month later the ocular examination was normal except for faint temporal conjunctival scar.

The patient was informed about the publication of his history and signed an inform consent.

## Discussion

Dirofilariasis infection sites are categorized into subcutaneous, lymphatic, and visceral sites. *D. repens* is mostly found in subcutaneous cases, while *D. immitis* is the main cause of pulmonary involvement. In the peripheral blood of natural host, the mature *Dirofilaria* turns into microfilariae. Conventionally, humans were supposed to be accidental hosts of dirofilaria. At the beginning of this accidental human infection cycle, the mosquitoes ingest the microfilariae from the natural hosts as mentioned before and they inject the larvae into human tissues. In this journey, most of the larvae cannot survive and for resisted larvae, it takes about 2 weeks to turn into microfilariae, which are the infectious stage of this parasite. After accidental injection of larvae to human body, it is commonly believed that although *Dirofilaria* larvae are capable of turning into adult worms these worms are fortunately sexually immature and as a result, at the end of this cycle no microfilariae can be created (12).

In 1885, an Italian ophthalmologist described the first case of eye infection by dirofilaria. *Dirofilaria* usually presents as a subcutaneous mass in ocular adnexa. Less often, it presents in the orbit or seldom, as a subconjunctival mass (12). *Dirofilaria* also can be found in Tenon’s layer (13), anterior chamber (14) and vitreous (10). There are numerous pathways for parasite to migrate across body. The microfilaria travels into the anterior chamber and vitreous cavity through blood. Sometimes the migration is continued to other parts of eye including lids and orbit via the subconjunctival and subcutaneous tissues. The only treatment for ocular dirofilariasis is complete removal of the parasite. Subconjunctival *Dirofilaria* is removed through an incision in the conjunctiva. The complete removal of parasite is essential in order to avoid an allergic reaction due to parasite remnants (12, 13). The reason for which the antihelminthic drugs seem not enough effective for treatment of this infection is inactivity of reproduction of *Dirofilaria* and single location of this parasite. (14) The rise of eosinophil count in blood analysis is one the earliest indicators of infection if the *Dirofilaria* is suspected. This rise of eosinophils returns to normal levels after complete removal of the parasite. These changes in peripheral blood cell count or localized presence of eosinophils in infected tissues, all suggest an allergic reaction caused by the inflammatory response induced by the nematode (12–14). Interestingly eosinophil count was in the normal range in our case. *Dirofilaria* is described by a quite large size, thick cuticle, and noticeable musculature. The species can be differentiated by their size, thickness of the cuticles, and the presence or absence of longitudinal ridges (15). However, this kind of morphological identification and description may not always be promising particularly in cases with incomplete section of the nematode or in cases with rare locations such as lymph nodes or viscera with associated inflammation. In these particular occasions, the best way for absolute species identification can be done by gene sequencing and gene comparison in the Gen Bank. (16)

Several surgical techniques are defined in the literature for retrieval of migratory worms in the periocular region (16–21). All recommended techniques are based on quick removal of a live worm while still detectable in operation field. Injection of lidocaine 1% with 1:100000 dilutions of epinephrine to restrain the worm and because of this paralysis, migration of the worm from its original site is avoided (16). There has been different hypothesis for the reason for recent increasing trend in prevalence of dirofilariasis.one of the most acceptable reasons is climate warming and growth in mosquito populations (22).

As we could not confirm the site of entry of the nematode into the subconjunctival space in our case and in the absence of any visible external trauma, we supposed that the infection was transmitted through mosquitoes via subcutaneous space. The interesting point was light sensitivity of the worm that caused overnight custodian to have photophobia during the daytime at the end of his work. The clinical point is that the worm has light sensitivity and this help the examiner to see the movement of worm under slit lamp light and surgeon should care about complete removal of the worm under operating microscope light because these excessive movements make the complete removal tough. Dirofilariasis is barely related to personal hygienic conditions because human is infected accidentally by blood sucking mosquitoes. Previous reports from subconjunctival *Dirofilaria* infection in Iran were the *D. repens*, however, this case is indicative of subconjunctival involvement by *D. immitis* (18–23).

## Conclusion

The growing rate of dirofilariasis infection shows the need for more awareness of medical consultants, proper preservation and identification of worms for making more precise diagnosis. Another important issue is watchful documentation of cases in order to determine distribution of infection all over the world. Up-to-date data on the incidence and prevalence of such diseases are important to those planning treatment and control of infections.

## Ethical considerations

Ethical issues (Including plagiarism, informed consent, misconduct, data fabrication and/or falsification, double publication and/or submission, redundancy, etc.) have been completely observed by the authors.
